# Coibamide A Induces mTOR-Independent Autophagy and Cell Death in Human Glioblastoma Cells

**DOI:** 10.1371/journal.pone.0065250

**Published:** 2013-06-06

**Authors:** Andrew M. Hau, Jeffrey A. Greenwood, Christiane V. Löhr, Jeffrey D. Serrill, Philip J. Proteau, Ian G. Ganley, Kerry L. McPhail, Jane E. Ishmael

**Affiliations:** 1 Department of Pharmaceutical Sciences, Oregon State University, Corvallis, Oregon, United States of America; 2 Department of Biochemistry & Biophysics, Oregon State University, Corvallis, Oregon, United States of America; 3 Department of Biomedical Sciences, Oregon State University, Corvallis, Oregon, United States of America; 4 MRC Protein Phosphorylation Unit, College of Life Sciences, University of Dundee, Dundee, Scotland, United Kingdom; Chinese University of Hong Kong, Hong Kong

## Abstract

Coibamide A is an *N*-methyl-stabilized depsipeptide that was isolated from a marine cyanobacterium as part of an International Cooperative Biodiversity Groups (ICBG) program based in Panama. Previous testing of coibamide A in the NCI *in vitro* 60 cancer cell line panel revealed a potent anti-proliferative response and “COMPARE-negative” profile indicative of a unique mechanism of action. We report that coibamide A is a more potent and efficacious cytotoxin than was previously appreciated, inducing concentration- and time-dependent cytotoxicity (EC_50_<100 nM) in human U87-MG and SF-295 glioblastoma cells and mouse embryonic fibroblasts (MEFs). This activity was lost upon linearization of the molecule, highlighting the importance of the cyclized structure for both anti-proliferative and cytotoxic responses. We show that coibamide A induces autophagosome accumulation in human glioblastoma cell types and MEFs via an mTOR-independent mechanism; no change was observed in the phosphorylation state of ULK1 (Ser-757), p70 S6K1 (Thr-389), S6 ribosomal protein (Ser-235/236) and 4EBP-1 (Thr-37/46). Coibamide A also induces morphologically and biochemically distinct forms of cell death according to cell type. SF-295 glioblastoma cells showed caspase-3 activation and evidence of apoptotic cell death in a pattern that was also seen in wild-type and autophagy-deficient (ATG5-null) MEFs. In contrast, cell death in U87-MG glioblastoma cells was characterized by extensive cytoplasmic vacuolization and lacked clear apoptotic features. Cell death was attenuated, but still triggered, in Apaf-1-null MEFs lacking a functional mitochondria-mediated apoptotic pathway. From the study of ATG5-null MEFs we conclude that a conventional autophagy response is not required for coibamide A-induced cell death, but likely occurs in dying cells in response to treatment. Coibamide A represents a natural product scaffold with potential for the study of mTOR-independent signaling and cell death mechanisms in apoptotic-resistant cancer cells.

## Introduction

There is high demand for new small molecules that can strategically target the dysregulated signaling pathways that underlie aggressive solid cancers such as glioblastoma. Glioblastoma multiforme (GBM), classed by the World Health Organization (WHO) as a high-grade IV astrocytoma-like tumor, is the most common malignant primary tumor of the central nervous system (CNS) and is associated with a particularly poor prognosis. Present therapeutic strategies have had little impact on the overall survival rate, with median patient survival times remaining at 14 to 19 months depending on the treatment regimen [1], [2], [3]. Collective efforts to classify the pathogenesis of gliomas have shown that GBM frequently harbors a signature of mutations that tend to attenuate the function of tumor suppressor genes, such as p53 and PTEN, or enhance activation of receptor tyrosine kinases such as epidermal growth factor receptor (EGFR) and platelet-derived growth factor receptor (PDGFR) (reviewed in [3],[4]). In turn, cell signaling driven by growth factors, such as the mitogen-activated protein kinase (MAPK) and phosphatidylinositol 3-kinase (PI3K) pathways, is dramatically enhanced. Together these aberrant signaling networks tend to promote cell survival and lend GBM a natural resistance to apoptosis, rendering conventional chemotherapeutic drugs that typically induce apoptosis ineffective for the treatment of this condition [3]. Consequently, there is a great need for new pharmacologic tools that cause cell death in glioblastoma and other apoptosis-resistant cancer cells.

As part of the ICBG program based in Panama, we previously reported the discovery of the *N*-methyl-stabilized cyclopeptide natural product coibamide A [5], named for its isolation from a marine cyanobacterium collected from the Coiba National Park, Panama. Coibamide A displayed an unprecedented profile in the National Cancer Institute 60 (NCI-60) human cancer cell line panel, showing sub-nanomolar potency as a growth inhibitory agent in many of the cell lines tested. Coibamide A was deemed “COMPARE-negative”, in that the pattern of cellular responses to coibamide A showed no significant correlation with any known agents in the NCI database. Initial characterization of the biological effects of coibamide A indicated that it did not interfere with tubulin or actin in cytoskeletal assays, or inhibit histone deacetylase activity. However, coibamide A was found to arrest cell cycle progression of MDA-MB-435 cancer cells at the G_1_ phase, indicative of the antiproliferative action of this cyanobacterial depsipeptide [5].

Coibamide A previously showed histological selectivity for several cancer cell types, with all six human glioma lines in the NCI’s *in vitro* cell panel showing high sensitivity [5]. When considered together, coibamide A produced mean cytostatic (GI_50_ and TGI) and cytotoxic (LC_50_) values in the CNS cell lines as follows: GI_50_ = 4.93±6.31 nM [log GI_50_, −8.60 (0.80)]; TGI = 3.86±1.32 µM [log TGI, −6.25 (3.12)] and LC_50_ values estimated as greater than 10 µM [log LC_50_, −5.00 (0)]. Given the potential of coibamide A as an experimental antitumor agent, the objective of the present study was to investigate the cytotoxic potential of coibamide A against glioma cells. We focused on two human glioblastoma cell lines: U87-MG, a well characterized, grade IV astrocytoma, and SF-295, representing one of the CNS tumor lines in the NCI-60 panel, and also utilized mouse embryonic fibroblasts (MEFs) derived from wild-type and genetically-modified animals. We report that coibamide A induces a rapid and sustained autophagic response via an mTOR-independent pathway, and is also a more potent and efficacious cytotoxic agent against human glioma cells than was previously appreciated. We show that autophagy is not required for coibamide A-induced cell death that, depending on the cellular context, can proceed via apoptotic or non-apoptotic pathways.

## Materials and Methods

### Reagents

The isolation of coibamide A and preparation of linearized coibamide A products has been described previously [5]. Purified coibamide A was reconstituted in 100% DMSO (2.0–2.3 mM), aliquoted and stored in amber borosilicate glass vials at −20°C for 3–6 months for use in biological studies. AZD 8055 was a kind gift from Professor Dario Alessi. Rapamycin, bafilomycin A1 and 3-(4,5-dimethylthiazol-2-yl)-2,5-diphenyltetrazolium bromide (MTT) were purchased from Sigma-Aldrich Corp. (St. Louis, MO). The caspase inhibitor Z-VAD-FMK was from EMD Millipore (Darmstadt, Germany). Cell culture grade DMSO was used as the vehicle for all treatments and never exceeded a final concentration of 0.1%. General reagents were purchased from Sigma-Aldrich Corp.

### Antibodies and Vital Stains

Primary and secondary antibodies were from commercial sources and used according to the recommendations of the supplier. For immunoblot analysis, antibodies to LC3-A/B (#4108), GAPDH (#2118), phospho-ULK1 Thr-757 (#6888), phospho-p70 S6 Kinase Thr-389 (#9205), p70 S6 Kinase (#9202), phospho-S6 ribosomal protein Ser-235/236 (#2211), total S6 ribosomal protein (#2217), phospho-4E-BP1 Thr-37/46 (#2855), 4E-BP1 (#9452), both full length and the 89 kDa fragment of PARP1 (#9532), the cleaved 89 kDa fragment of PARP1 (#9544), and caspase-3 (#9662) were from Cell Signaling Technology, Inc. (Danvers, MA). The antibody to EGF receptor (#sc-03) was from Santa Cruz Biotechnology, Inc., (Santa Cruz, CA), alpha-tubulin (#cp-06) was from EMD Millipore and antibodies to ULK1 (#A7481) and actin (#A5060) were from Sigma-Aldrich. Secondary antibodies coupled to horseradish peroxidase (HRP) were from EMD Millipore or Thermo Fisher Scientific Inc. (Waltham, MA). For immunocytochemistry of endogenous LC3 we used a mouse monoclonal antibody (#M152-3B) from MBL International (Woburn, MA), with an Alexa Fluor 594-coupled goat anti-mouse secondary antibody (#A-11005) from Molecular Probes-Invitrogen (Life Technologies, Grand Island, NY). ProLong Gold mounting medium with DAPI counterstain and monodansylcadaverine (MDC) were also from Invitrogen.

### Cell Culture

Human U87-MG glioblastoma cells and mouse neuroblastoma Neuro-2A cells were purchased from the American Type Culture Collection (ATCC, Manassas, VA). Human SF-295 and U251 glioblastoma cells were obtained from the National Cancer Institute (NCI) cell line repository (Frederick, MD). ATG5-null MEF cells were a kind gift from Dr. Noboru Mizushima, Tokyo Medical and Dental University [6], and Apaf-1-null MEFs a kind gift from Dr. Tak Mak at the University of Toronto [7]. U87-MG and U251 cells were maintained in Modified Eagle’s Medium (MEM), and MEFs in Dulbecco’s Modified Eagle’s Medium (DMEM; Mediatech Inc., Manassas, VA), supplemented with 10% fetal bovine serum (FBS; HyClone, Logan, UT), L-glutamine (2 mM) and 1% penicillin/streptomycin (Mediatech Inc.). Neuro-2A and SF-295 cells were cultured in RPMI-1640 medium supplemented with L-glutamine (2 mM), sodium pyruvate (1 mM), sodium bicarbonate (1.5 g/L), and penicillin/streptomycin and 10% FBS. All cell lines were routinely maintained in a humidified chamber at 37°C with 5% CO_2_.

### Cell Viability Assays

For MTT, Trypan Blue exclusion and LDH assays, cells were seeded into 96-well flat-bottom plates (BD Biosciences, Franklin Lakes, NJ) and maintained overnight before treatment as indicated. MTT assays were based on a method described by Mosmann [8]. MTT was dissolved in phosphate-buffered saline (PBS) and added to each well at a final concentration of 0.5 mg/mL. Plates were allowed to incubate for 2 h at 37°C, after which time the medium was removed and the purple formazan product solubilized with DMSO (100 µL). The optical density of each well was determined at 550 nm using a Synergy HT microplate reader with Gen5 software (Bio-Tek Instruments, Inc, Winooski, VT). Cytotoxicity was determined in at least three independent experiments. The viabilities of vehicle-treated cells ranged from 97.6 to 104.6% relative to untreated cells, thus the viability of untreated control cells was defined as 100%. For Trypan Blue exclusion assays, the medium was aspirated from each well at the time points indicated and the cells detached with 0.25% trypsin-EDTA (20 µL). Cells were then collected by the addition of serum-free DMEM (40 µL) mixed with Trypan Blue and the viable cells counted. LDH activity was measured as previously described [9] using a CytoTox 96® Non-Radioactive Cytotoxicity Assay (Promega) according to the manufacturer’s instructions.

### Caspase Activity Assay

Cells were seeded in 96-well white-walled, clear-bottom plates (Greiner Bio-One, Monroe, NC) and treated with or without coibamide A (10 nM to 300 nM) for the times indicated. Caspase-3,7 activity was measured using a luminescence-based Caspase-Glo® 3/7 assay (Promega). The Caspase-Glo® reagent, which also serves to lyse the cells, was added directly to each well (50 µL) and the resulting luminescence was measured every 10 min for 2 h using a Synergy HT microplate reader.

### Autophagy Assays

To assess autophagic flux, cells were incubated with coibamide A (30 nM) or vehicle (DMSO) for 4 h, in the presence (+) or absence (-) of bafilomycin (10 nM) added for the final hour of treatment. For starvation-induced autophagy, cell monolayers were washed twice and then incubated in Earle’s Balanced Salt Solution (GIBCO-EBSS, Life Technologies) for 4 h.

### Epidermal Growth Factor (EGF) Receptor Degradation Assay

EGF receptor degradation assays were based on a method described previously [10]. On the day of the experiment, U87-MG cell monolayers were rinsed twice and then incubated in serum-free medium for 2.5 h. Coibamide A (30 nM) or DMSO (control) was then added to the plates for a further 1 h incubation. At time zero, the medium was replaced with serum-free medium plus EGF (100 ng/mL), and cycloheximide (25 µg/mL), with either vehicle (DMSO), coibamide A (30 nM), or bafilomycin A1 (100 nM). Cells were returned to the incubator until the indicated times when they were lysed and processed for immunoblot analysis.

### Cell Lysis and Immunoblot Analysis

At the end of treatment cells were placed on ice, washed with PBS and lysed in ice-cold buffer containing 50 mM Tris-HCl, pH 7.5, 1 mM EDTA, 1 mM EDTA, 1% Triton X-100, 0.27 M sucrose, 50 mM sodium fluoride, 1 mM sodium orthovanadate, 5 mM sodium pyrophosphate, 1 mM PMSF and 1 mM benzamidine. For preparation of lysates from detached cells, the culture medium was removed and cells collected by gentle centrifugation at 800×*g* for 5 min. The cell pellet was rinsed in PBS and then resuspended in the same lysis buffer; adherent cells from these plates were processed as above. All cell lysates were cleared by centrifugation at 16,000×*g* for 20 min at 4°C and the protein concentration determined using the bicinchoninic acid (BCA) method according to the manufacturer’s recommendations (Thermo Fisher Scientific Inc.). For immunoblot analysis, cell lysates were adjusted for protein content and equal amounts (20 µg) separated by SDS-PAGE. Proteins were immobilized onto either Hybond-ECL (Amersham, Piscataway, NJ) or PVDF (Thermo Fisher Scientific) membranes. Membranes were blocked in 5% (w/v) non-fat dry milk in 50 mM Tris-HCl, pH 7.4, 150 mM NaCl (TBS) plus 0.1% Tween-20 (TBS-Tween), and then incubated for 16 h at 4°C with the appropriate primary antibody in 5% (w/v) bovine serum albumin (BSA). The following day, membranes were washed in TBS-Tween (3×10 min) then incubated with the appropriate HRP-conjugated secondary antibody for 1 h at room temperature. Membranes were washed again in TBS-Tween (4×5 min), and proteins revealed using an enhanced chemiluminescence (ECL) reagent.

### Fluorescence Microscopy

For live imaging cells were grown on MatTek 35 mm glass-bottom culture dishes (MatTek Corp., Ashland, MA) in complete medium in the presence or absence of coibamide A (20 nM), rapamycin (100 nM) or vehicle (DMSO) as indicated. The procedure for vital staining with MDC was adapted from procedures described by others [11],[12]. Briefly, cells were rinsed in Hank’s Buffered Salt Solution (HBSS), before incubation with MDC (50 nM) in HBSS for 10 min at 37°C. The MDC solution was then removed, the cells rinsed in HBSS and maintained in HBSS for immediate observation using a Zeiss Axiovert S100TV fluorescent microscope with a Hoechst filter set and MetaMorph imaging software. Images were captured using a Photometrics CoolSNAP HQ CCD camera.

For immunocytochemistry of microtubule-associated protein light chain 3 (LC3), cells were grown and treated on glass coverslips as indicated. At the end of the treatment cells were washed twice in PBS and fixed in 3.7% formaldehyde in PBS for 20 min at room temperature. Cells were then washed twice and incubated in DMEM/10 mM HEPES (pH 7.4) for 10 min to quench the formaldehyde. After two washes in PBS, cells were permeabilized with 0.2% NP-40 in PBS for 3 min at room temperature. Cells were washed twice, to remove the NP-40, and then blocked for 15 min with 1% BSA in PBS (BSA/PBS). Cells were incubated for 1 h with anti-LC3, diluted in 1∶500 in BSA/PBS. The coverslips were washed in BSA/PBS (3×10 min) and then incubated with an Alexa Fluor-conjugated secondary antibody, diluted 1∶500 BSA/PBS, for 30 min at room temperature. Coverslips were again washed in BSA/PBS (3×10 min), mounted onto glass microscope slides and cells observed using a Nikon Eclipse Ti-S microscope equipped with a Nikon DS-digital CCD camera. Images were analyzed using NIS-Elements imaging software (Nikon Instruments).

### Transmission Electron Microscopy (TEM)

The preparation of cells for ultrastructural analysis was adapted from the method of Shingu et al [13]. Briefly, U87-MG cells were treated with or without coibamide A (20 nM) or vehicle (DMSO) as indicated. Cells were washed three times with PBS, collected by trypsinization, and fixed in a solution containing 2.5% glutaraldehyde/1% paraformaldehyde in cacodylate buffer, then post-fixed with 1% buffered osmium tetroxide for 30 min. After dehydration in acetone, infiltration and embedding in Spurr’s medium, the cell samples were polymerized for 24 h at 65°C. Samples were cut into ultrathin sections with a Sorvall MT-2 microtome (Thermo Fisher Scientific), and then stained with uranyl acetate and lead citrate in a Leica EM Stainer (Leica, Deerfield, IL). Sections were examined with a Phillips CM12 scanning transmission electron microscope (Phillips, Andover, MA) at an accelerating voltage of 80 kV.

### Data Analysis

Concentration-response relationships were analyzed using Graphpad Prism Software (Graphpad Software Inc., San Diego, CA), and EC_50_ values derived using nonlinear regression analysis fit to a logistic equation. For Trypan Blue exclusion studies, time-response relationships were analyzed with the same software and fit to a standard exponential growth curve. For analysis of EGFR degradation and LC3 flux by immunoblot, the intensity of EGFR or LC3 signals was normalized to the intensity of tubulin and quantified relative to the control using Image J software (rsbweb.nih.gov/ij). For quantitation of mean LC3 puncta per cell by immunocytochemistry, images were captured from two random fields of view per coverslip and LC3 puncta (Alexa Fluor 594) counted with NIS-Elements software and compared to the total number of cells (DAPI). Statistical significance of data derived from MTT viability assays, LDH efflux and caspase activity was performed using a one-way analysis of variance (ANOVA) followed by a student’s *t*-test comparing untreated controls and treatment groups. *P*-values of 0.05 or less were considered statistically significant.

## Results

### Cytotoxic Effect of Coibamide A on Human Glioblastoma Cells

**Figure 1:**
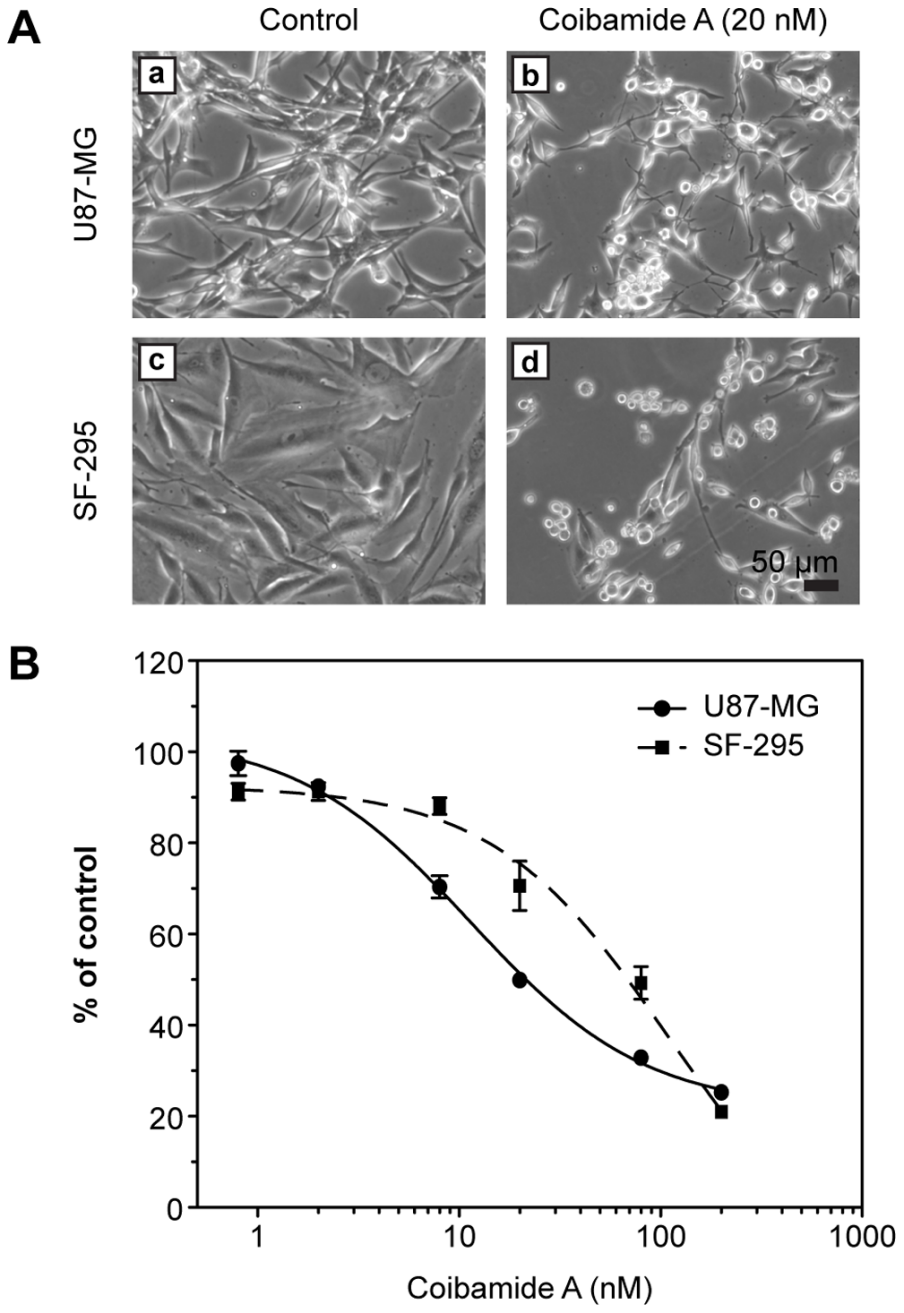
Cytotoxic effect of coibamide A on human glioblastoma cells. (**A**) Cell death in U87-MG (upper panels) and SF-295 (lower panels) glioma cells after a 3-day exposure to coibamide A (20 nM). Cell death was documented by morphological evaluation of vehicle-(DMSO; panels a and c) or coibamide A-treated (panels b and d) cells using light microscopy. (**B**) Concentration-response profile for coibamide A-induced cytotoxicity in U87-MG and SF-295 cells. Glioma cells were treated with increasing concentrations of coibamide A (2.3 to 230 nM) for 3 days. Cytotoxicity was determined by MTT assay with the viability of control cells defined as 100%. Dose-response data represent mean viability ± SE (n = 3 wells per treatment) from a comparison that was repeated in at least four independent experiments.

Coibamide A produced concentration- and time-dependent cell death in human U87-MG and SF-295 glioblastoma cells. Overt cytotoxicity was not evident within the first 24 to 36 h of coibamide A exposure. When treatment times were extended however, coibamide A caused progressive rounding and detachment of both glioma cell lines from the culture plates. As illustrated in figure 1A, both U87-MG and SF-295 cells showed reduced proliferation and significant changes in morphology by 72 h of exposure to coibamide A (20 nM) relative to vehicle-treated cultures. Coibamide A-induced cytotoxicity was concentration-dependent with EC_50_ values of 28.8±8.4 nM and 96.2±23 nM for U87-MG and SF-295 cells, respectively (Fig. 1B).

**Figure 2:**
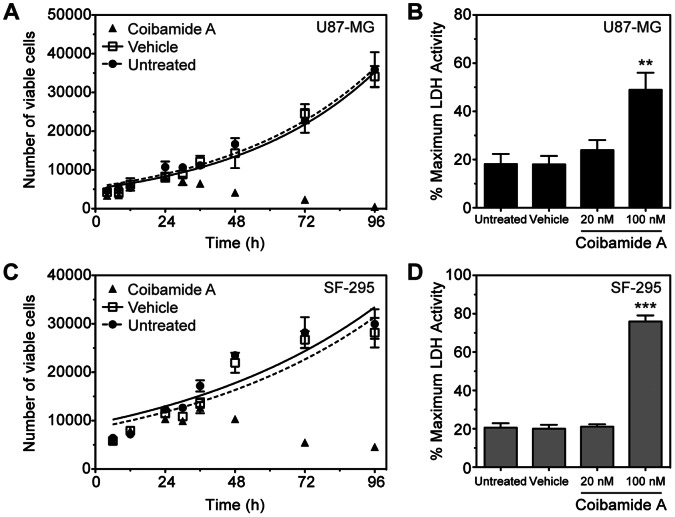
Coibamide A induces a time- and concentration-dependent decrease in plasma membrane integrity. (**A**) Trypan Blue exclusion test of cell viability in U87-MG cultures treated with or without coibamide A (20 nM) or vehicle (DMSO) over 4 days. (**B**) Concentration-response relationship of LDH efflux in U87-MG cells treated with or without coibamide A or vehicle for 4 days (**C**) Trypan Blue exclusion test of cell viability and (**D**) concentration-response relationship of LDH efflux in SF-295 cells treated with or without coibamide A or vehicle (as above) for 4 days. Trypan Blue exclusion profiles represent mean cell counts ± SD of a representative time course performed in triplicate and repeated at least three times in each cell line. Histograms represent mean LDH efflux ± SE of at least three independent experiments performed in triplicate (**P<0.01 and ***P<0.001, coibamide A *vs.* untreated control).

To examine the temporal nature of coibamide A-induced toxicity, we used Trypan Blue exclusion to monitor cell viability for up to 96 h after treatment with a fixed concentration of coibamide A (20 nM). U87-MG cells showed no change in their ability to exclude Trypan Blue until between 24 and 36 h after exposure to coibamide A, but thereafter the number of viable cells began to decrease in a time-dependent manner (Fig. 2A). Few coibamide A-treated U87-MG cells remained viable after 96 h relative to untreated or vehicle-treated cultures. The observed loss of membrane integrity over time prompted us to also investigate the potential of coibamide A to induce release of LDH, a biomarker of membrane damage. For these studies the culture medium was collected from coibamide A-treated cells and the LDH content of the medium determined relative to total LDH in the remaining adherent cells. We found no significant increase in LDH leakage in cultures treated with 20 nM coibamide A, however LDH was significantly elevated in cultures exposed to 100 nM coibamide A (Fig. 2B). SF-295 glioblastoma cells showed the same pattern of cytotoxicity in that the number of viable cells decreased in a time-dependent manner after 24 h (Fig. 2C) and LDH was elevated significantly in exposed cultures (Fig. 2D). Together these results indicate that coibamide A induces both time- and concentration-dependent cytotoxicity in human glioma cells.

### Coibamide A Induces Activation of Caspase-3/7 and Apoptosis in a Cell Type-specific Manner

**Figure 3:**
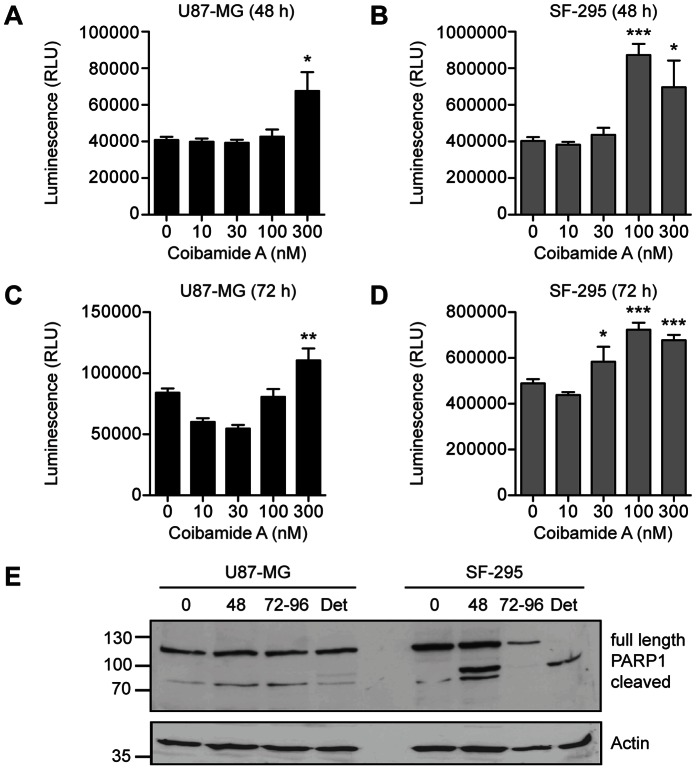
Coibamide A induces activation of caspase-3,7 and apoptosis in a cell type-specific manner. Concentration-response relationship for coibamide A-induced caspase-3,7 activation in (**A**) U87-MG and (**B**) SF-295 cells exposed to coibamide A (10–300 nM) or vehicle (DMSO) for 48 h. Coibamide A-induced caspase-3,7 activation in (**C**) U87-MG and (**D**) SF-295 cells exposed to coibamide A (10–300 nM) for 72 h. Caspase-3,7 activity was measured at the end of each treatment using a luminescence-based assay coupled with automated luminescence detection. Histograms represent mean ± SE of up to four independent determinations (**P<0.01 and ***P<0.001, coibamide A vs. vehicle control). (**E**) Immunoblot analysis of PARP1 in coibamide A-treated (100 nM) U87-MG and SF-295 cells lystates. Adherent and detached (Det) cells were harvested over 4 days and examined for expression of the large 89 kDa fragment of PARP1, a marker of apoptosis, plus actin as a loading control. Immunoblot is representative of an experiment repeated at least three times with similar results.

To provide some insight into the potential mechanism of coibamide A-induced cell death we tested the ability of coibamide A to activate the downstream effector caspases of the major apoptotic signaling pathways: caspase-3 and caspase-7. Coibamide A induced activation of caspase-3/7 in U87-MG and SF-295 glioma cells over a time frame consistent with loss of viability, however the activation profile for each cell line was distinct. Although U87-MG cells were more sensitive than SF-295 cells to coibamide A-induced cell death, as determined by MTT cell viability assays (Fig. 1B), relatively high concentrations of coibamide A were required to induce late activation of caspase-3/7 in these cells (Fig. 3A and 3C). In contrast, coibamide A was a much more potent and effective activator of caspase-3/7 in the SF-295 glioma cell line (Fig. 3B and 3D). We therefore collected attached and detaching coibamide A-treated cells over the course of a 96 h exposure period and analyzed cell lysates for expression of PARP1, a major downstream target of caspase-dependent apoptosis and several alternative cell death pathways [14]. An 89 kDa band corresponding to the caspase 3-cleaved form of PARP1 was readily detected by 48 h indicative of apoptotic cell death in SF-295 cells, whereas only trace levels of this fragment were observed in late, detaching U87-MG cell lysates (Fig. 3E).

**Figure 4:**
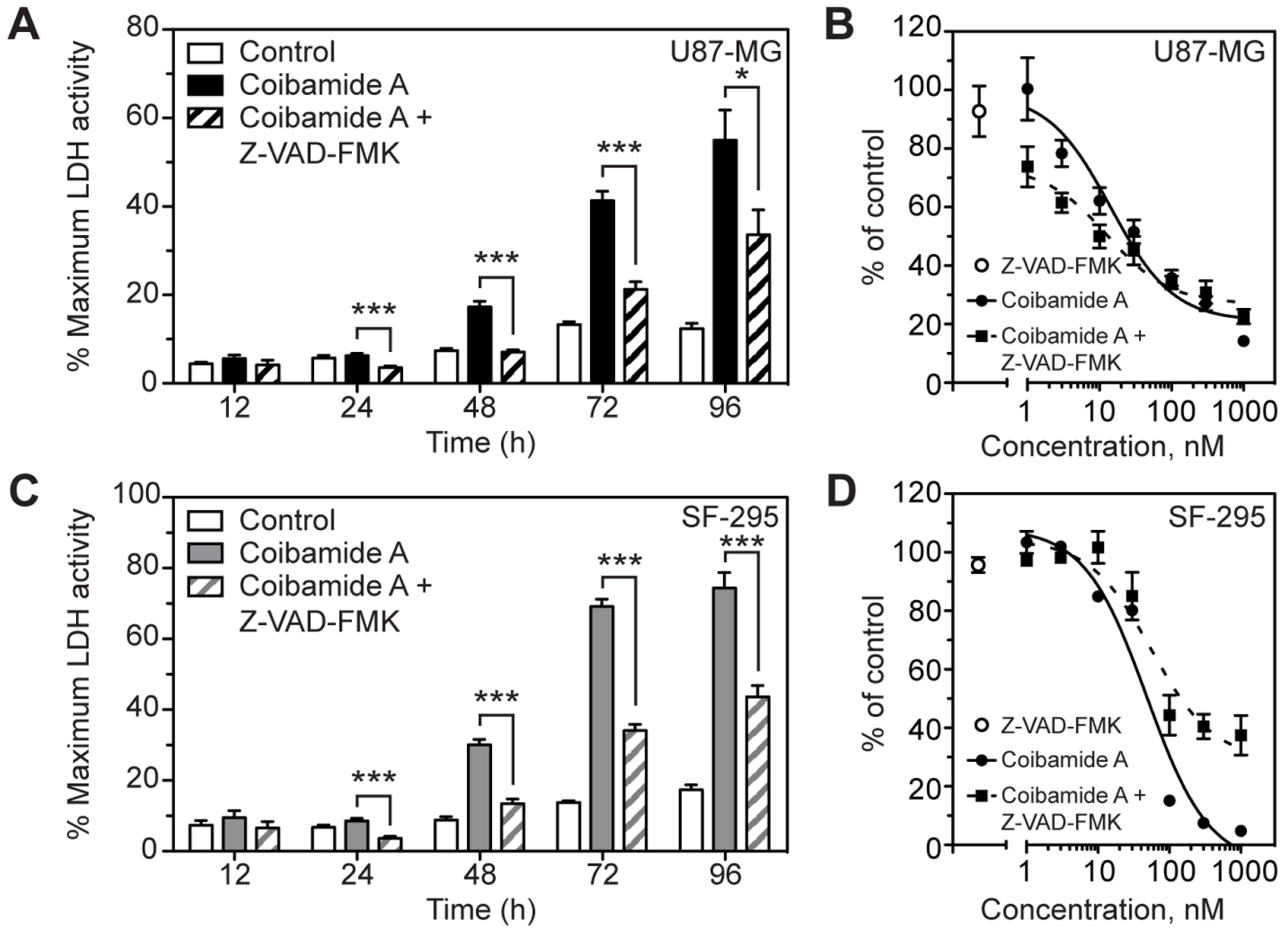
The broad-spectrum caspase inhibitor Z-VAK-FMK inhibits LDH release but does not prevent coibamide A-induced cell death. (**A**) Time course of LDH efflux from U87-MG cells over a 4 day exposure to coibamide A (300 nM) in the presence or absence of Z-VAD-FMK (100 µM). (**B**) Concentration-response relationship of coibamide A-induced cell death in the presence or absence of Z-VAD-FMK (100 µM). (**C**) Time course of LDH efflux and, (**D**) concentration-response relationship of coibamide A-induced cell death in SF-295 cells measured in the presence or absence of Z-VAD-FMK (100 µM). Histograms represent mean LDH efflux ± SE of three independent experiments performed in triplicate (**P<0.01 and ***P<0.001, coibamide A *vs.* control). Cytotoxicity was determined by MTT assay after 3 days of exposure to coibamide A (300 nM) with the viability of control cells defined as 100%; cell viability in the presence of Z-VAD-FMK alone (100 µM) is represented by an open circle. Dose-response data represent mean viability ± SE (n = 3 wells per treatment) from a comparison that was repeated in three independent experiments.

Given the potential for coibamide A to induce cell type-specific caspase-3/7 activation, we investigated the influence of pharmacologic inhibition of caspase activity with the broad-spectrum caspase inhibitor Z-VAD-FMK. Treatment with Z-VAD-FMK (100 µM) alone produced no significant change in the viability of either SF-295 (96±3%) or U87-MG (94±8%) cells relative to control cells as determined by MTT cell viability assays (Fig. 4). Co-treatment of cells with coibamide A and Z-VAD-FMK produced a significant inhibition of coibamide A-induced LDH efflux from U87-MG cells (Fig. 4A), however this caspase activity appeared to be non-essential as the inhibitor failed to protect against cell death and instead tended to exacerbate coibamide A-induced cytotoxicity in U87-MG cells in the presence of low concentrations (<30 nM) of coibamide A (Fig. 4B). Z-VAD-FMK was effective at suppressing coibamide A-induced LDH release from SF-295 cells (Fig. 4C), but in this case also afforded some protection from coibamide A-induced cell death (Fig. 4D). The addition of Z-VAD-FMK attenuated the cytotoxic efficacy of coibamide A with 40% of the SF-295 population remaining viable after 72 h in response to relatively high concentrations (100 nM to 1µM) of coibamide A. Taken together, these findings indicate that coibamide A can induce apoptosis via activation of a classic caspase-3-dependent pathway in SF-295 cells, but can also effectively trigger cell death via an alternate pathway in U87-MG glioma cells when caspase activity is inhibited.

### Linearized forms of Coibamide A Lack Cytotoxic Potential

**Figure 5:**
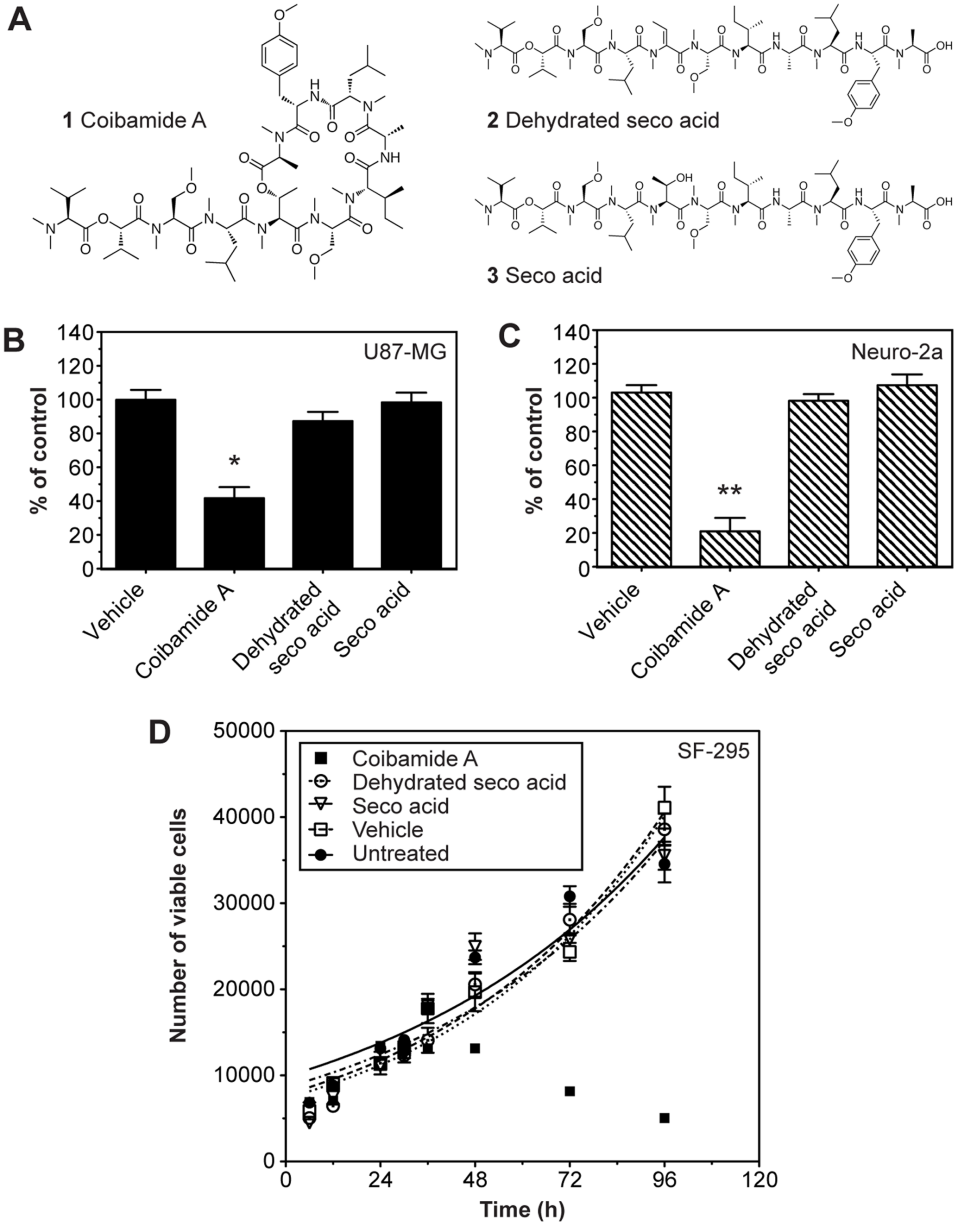
Linearized analogs of coibamide A are not cytotoxic. (**A**) Structures of coibamide A (**1**), and two linearized derivatives: dehydrated seco acid (**2**), and seco acid (**3**). (**B** and **C**) Comparison of cell viability in human U87-MG glioblastoma and mouse Neuro-2A neuroblastoma cells, respectively, following exposure to the coibamide A (20 nM) parent structure, linear forms **2** and **3** (both 30 µg/mL, ∼230–260 nM), or vehicle (DMSO) for 4 days. (**D**) Trypan Blue exclusion test of cell viability over 4 days in SF-295 cultures treated with or without the coibamide A (20 nM), linear forms **2** and **3** (both 30 µg/mL, ∼230–260 nM), or vehicle (DMSO). Histograms represent mean cell viability ± SE of at least three independent MTT assays performed in triplicate (*P<0.05 and ** *P<0.01*, coibamide A *vs.* control). Trypan Blue exclusion profile represents mean cell counts ± SD of a representative time course performed in triplicate that was repeated three times with similar results.

Coibamide A is a lariat depsipeptide that features a highly methylated 22-membered macrocycle and pseudo-tetrameric side chain (**1**; Fig. 5A). In order to confirm the cyclization point of coibamide A, the natural product was originally subjected to base hydrolysis, producing four linear derivatives [5]. The two full-length linearized analogs were designated coibamide dehydrated seco acid (**2**; Fig. 5A) and coibamide seco acid (**3**; Fig. 5A). Towards the goal of determining the coibamide A pharmacophore, we studied the biological activities of **2** and **3** in U87-MG and SF-295 glioblastoma, and mouse Neuro-2A neuroblastoma cells (Fig. 5B to 5D). Both MTT and Trypan Blue exclusion viability assays were used to monitor the extent and pattern of toxicity in the three tumor lines for up to 96 h after treatment with coibamide A (20 nM), or 0.3 µg/mL of linear derivatives **2** or **3** (∼230–260 nM). In the native form, coibamide A produced a significant, time-sensitive reduction in the viability of U87-MG, SF-295 and Neuro-2A cells relative to vehicle or untreated control cultures (Fig. 5B to 5D). However, no significant change in the viability of these three cell types was observed in response to treatments with the linearized derivatives **2** and **3** (Fig. 5B to 5D). U87-MG cells remained 87% and 98% viable relative to vehicle-treated cells after 96 h exposure to **2** and **3**, respectively (Fig. 5B). Similarly, the viability of Neuro-2A neuroblastoma cells was 98% and 107% following exposure to **2** and **3**, respectively (Fig. 5C). When examined by Trypan Blue exclusion (shown for SF-295 cells in Fig. 5D), those cultures treated with **2** or **3** were found to be indistinguishable from either untreated or vehicle-treated control cultures.

### Coibamide A Induces Autophagosome Accumulation in Apoptotic-resistant U87-MG Cells

**Figure 6:**
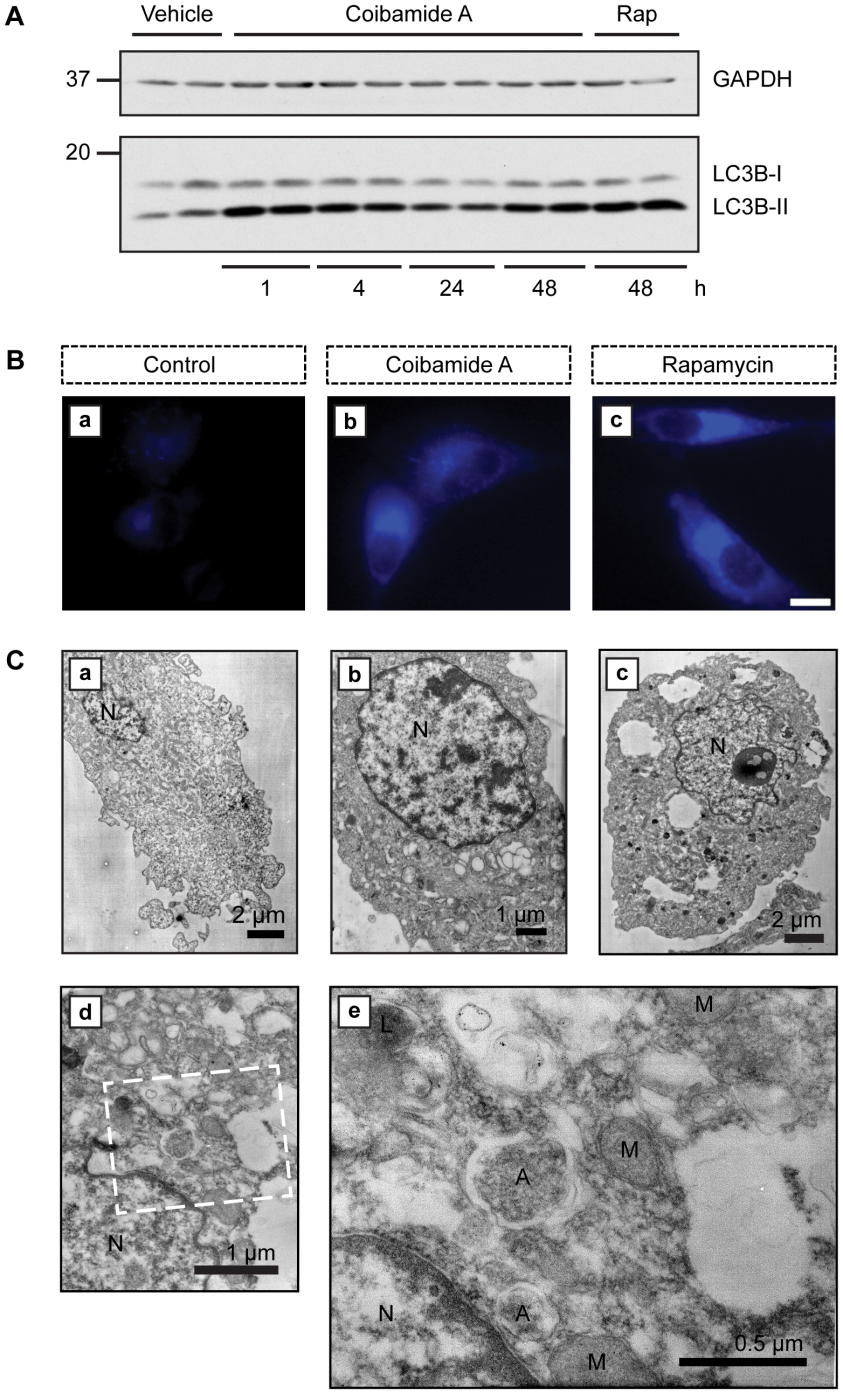
Coibamide A induces biochemical and morphological features of autophagy. (**A**) Time course of endogenous LC3 expression in U87-MG cell lysates treated with coibamide A (20 nM) for up to 48 h, relative to vehicle (DMSO) or rapamycin- (100 nM)-treated control cells. Cell lysates were subjected to immunoblot analysis with an antibody recognizing both forms of LC3: cytosolic LC3-I and the autophagosome-associated LC3-II. Anti-GAPDH was used as a loading control. Data are representative of a time course repeated at least three times. (**B**) Monodansylcadaverine (MDC) staining of U87-MG cells treated with vehicle (DMSO; panel a), coibamide A (20 nM; panel b), or rapamycin (100 nM; panel c) for 48 h. After treatment cells were stained with MDC (50 mM) for 10 min. Images are representative of a pattern of staining observed in at least three independent experiments. Scale bar = 10 µm. (**C**) Electron micrographs of adherent U87-MG cells treated with or without coibamide A (20 nM). Ultrastructure of representative control (panel a) and treated U87-MG cells at 16 (panel b) and 48 h (panels c to e). By 48 h coibamide A-treated cells showed empty vacuoles that could be distinguished from autophagosomes (A) with a discernible double membrane. The nucleus (N), mitochondria (M), lysosome (L) and scale bars are indicated.

To investigate coibamide A-induced signaling further, we studied the potential of coibamide A to induce autophagy using biochemical and morphological criteria. LC3, also termed autophagy-related (ATG) protein 8, is commonly used as a biochemical marker of autophagy because it is directly recruited to autophagosomes [15],[16]. LC3 can be detected as two distinct bands by immunoblot analysis: LC3-I is the cytosolic form that migrates more slowly than LC3-II, which is recruited to the membrane of autophagosomes. We analysed the expression of endogenous LC3 in lysates derived from adherent U87-MG cells that had been treated with coibamide A for up to 48 h. By 1 h we observed a clear increase in LC3-II expression in coibamide A-treated cells relative to control (Fig. 6A). This increase in LC3-II expression was generally sustained through 48 h and comparable to that observed with the macrolide natural product rapamycin (Fig. 6A), a known inducer of autophagy in glioblastoma cells [12]. We next stained treated cells with MDC, a fluorescent marker used as a probe for mature autophagosomes and lysosomes [17]. Vital staining of coibamide A-treated U87-MG cells with MDC revealed increased accumulation of the dye relative to control cells (Fig. 6B, panels a and b). Furthermore, we found this effect to be qualitatively indistinguishable from cytosolic MDC accumulation observed for cells treated with rapamycin (Fig. 6B, panels b and c). We also utilized TEM to visualize the ultrastructure of adherent U87-MG cells following exposure to coibamide A at 16 and 48 h (Fig. 6C). These studies revealed no evidence of nuclear condensation and segregation, or cytoplasmic shrinking expected of cells undergoing apoptosis. The nuclear membrane frequently had an undulating character to it, but was discernable as an intact double-membrane. Cells exposed to coibamide A for 16 h showed small electron lucent vacuoles that were absent in control cells (Fig. 6C, panels a and b). Empty vacuoles were also observed at 48 h by TEM, with 20% of cells containing one or more large (>2µm) cytoplasmic vacuole (Fig. 6C, panel c). At higher magnification (Fig. 6C, panels d and e) empty vacuoles lacking an organized double-membrane could be clearly distinguished from autophagosomes (A), that were delineated by a smooth double-membrane containing cellular material, intact mitochondria (M) and more electron-dense lysosomal (L) structures.

**Figure 7:**
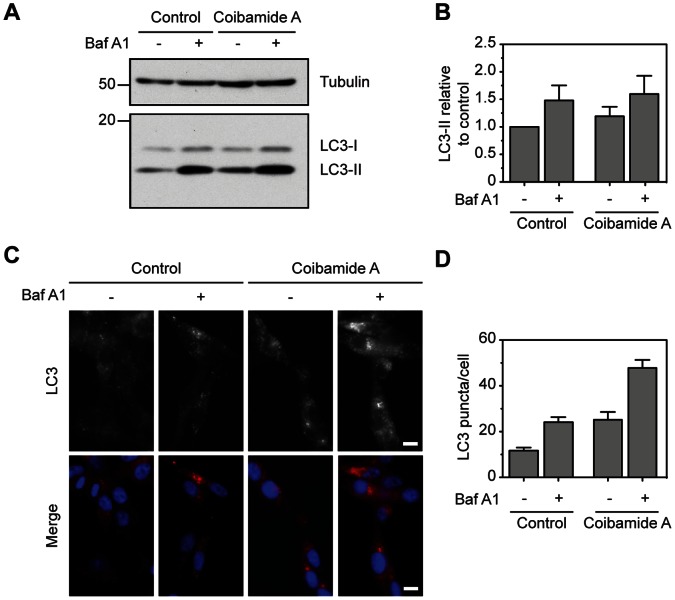
Coibamide A induces autophagosome accumulation in U87-MG cells. (**A**) Immunoblot analysis of endogenous LC3 in U87-MG cell lysates treated with vehicle (control) or coibamide A (30 nM) for 4 h, each in the presence or absence of bafilomycin A1 (10 nM) for the final 1 h of treatment. (**B**) Quantitation of LC3-II levels as shown in panel A. Histogram represents mean LC3-II intensity, normalized to tubulin, from immunoblots generated in three independent experiments. (**C**) Immunocytochemistry of U87-MG cells treated with vehicle (control) or coibamide A (30 nM) for 4 h, each in the presence of absence of bafilomycin (10 nM) for the final 1 h of treatment. Cells were stained for endogenous LC3 (red) and DAPI (blue). Scale bar, 10 µm. (**D**) Quantitation of mean LC3 puncta per cell (n = 32 cells) from three independent experiments.

As an apparent increase in autophagosome number could be the result of increased formation, or decreased degradation of autophagosomes, we employed a standard technique to assess autophagic flux in U87-MG cells in response to coibamide A exposure. For these studies cells were treated with or without coibamide A for 4 h, in the presence or absence of the macrolide natural product bafilomycin A1 for the last hour of treatment. Bafilomycin A1 is a potent inhibitor of the vacuolar-type H(+)-ATPase that is required for fusion of autophagosomes with lysosomes and thus blocks lysosomal degradation of LC3 [18],[19]. Even under control conditions, bafilomycin treatment induced a strong accumulation of LC3-II, indicating that these cells have a high basal autophagic flux (Fig. 7A and B). Similarly, bafilomycin also resulted in accumulation of LC3-II in cells treated with coibamide, confirming that coibamide is not blocking autophagosomal turnover. Given the high basal flux in these cells it was hard to determine the degree of coibamide-induced autophagy using the western blot-based assay. We therefore employed immunofluorescence to look at endogenous LC3 localization to autophagosomes. We found that coibamide A treatment induced a punctate distribution of LC3, particularly in the peri-nuclear region of the cytoplasm, and this was consistently enhanced by the addition of bafilomycin A1 (Fig. 7C and 7D). When comparing with control cells, coibamide resulted in an approximate two-fold increase in the number of LC3 puncta per cell, seen in the presence or absence of bafilomycin A1 (Fig. 7C and 7D). Taken together, the MDC staining, electron microscopy and LC3 flux, by western and immunofluorescence, strongly imply coibamide is inducing autophagy in U87-MG cells.

### Coibamide A does not Change the Rate of EGF Receptor Degradation

**Figure 8:**
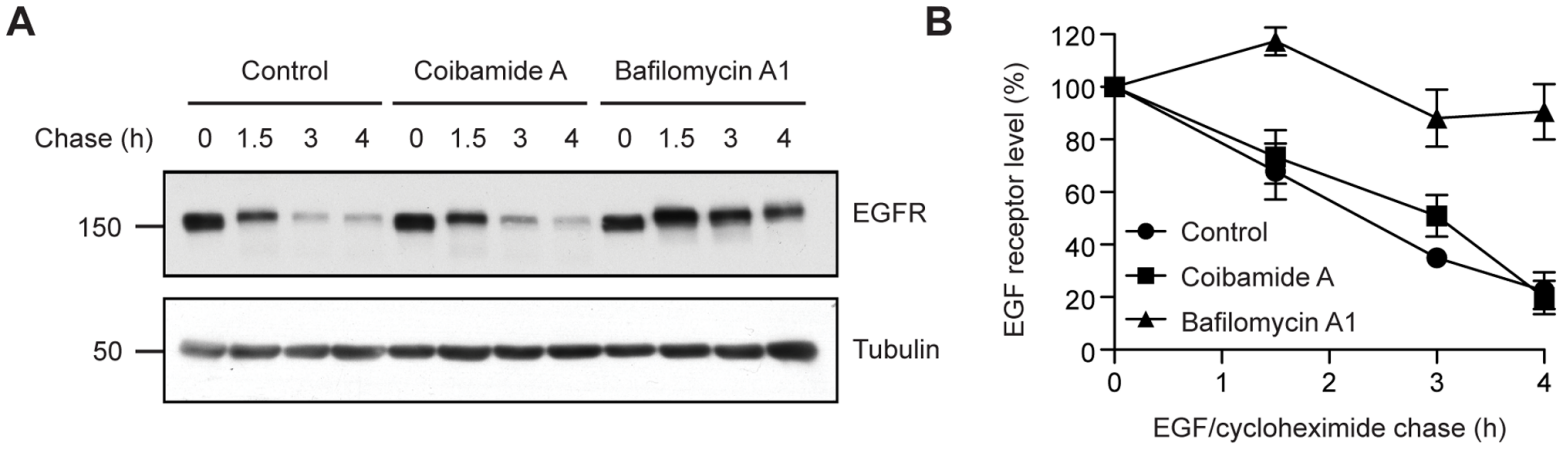
Coibamide A does not block ligand-induced degradation of EGFR. (A) Immunoblot analysis of EGFR expression up to 4 h after the addition of ligand (EGF) in the presence of vehicle (control), coibamide A (30 nM) or bafilomycin A1 (100 nM). On the day of the experiment U87-MG cells were starved of serum, to promote membrane localization of EGFR, and at t = 0 were chased with EGF (100 ng/mL) and cycloheximide (25 µg/mL), with or without coibamide A, or bafilomycin A1. Cells were lysed at the times indicated and processed for immunoblot analysis. (B) Quantitation of ligand-induced EGFR degradation from three independent experiments as described in A. Graph represents % EGFR expression, following the addition of EGF, as a function of incubation time in the presence of vehicle, coibamide A or bafilomycin A1. EGFR intensity was normalized to tubulin and determined relative to EGFR level at t = 0.

Autophagy requires a coordinated series of specific endomembrane fusion events to traffic the contents of autophagosomes to the lysosomes for degradation. We therefore assessed the potential of coibamide A to interfere with a major endocytic-lysosomal pathway by studying turnover of endogenous EGFR. For these studies, U87-MG cells were serum-starved to promote localization of EGFR to the plasma membrane and were then chased in the presence of EGF (100 ng/mL), to induce internalization of EGFR, plus cycloheximide (25 µg/mL) to inhibit protein synthesis. In the presence of coibamide A, ligand-induced EGFR degradation progressed at a rate that could not be distinguished from vehicle-treated control cells (Fig. 8A and 8B). This finding was in contrast to U87-MG cells cultured in the presence of bafilomycin A1. Bafilomycin A1 dramatically inhibited endocytosis-mediated degradation of EGFR over the time course of these experiments (Fig. 8A and B), in a manner consistent with its ability to block lysosomal degradation of EGFR. Taken together our findings indicate that the autophagic-lysosomal pathway remains functional in U87-MG cells in the presence of coibamide A and that coibamide A does not produce a global block in endocytosis.

### Coibamide A Induces a Conventional form of Autophagy in Mouse Embryonic Fibroblasts

**Figure 9:**
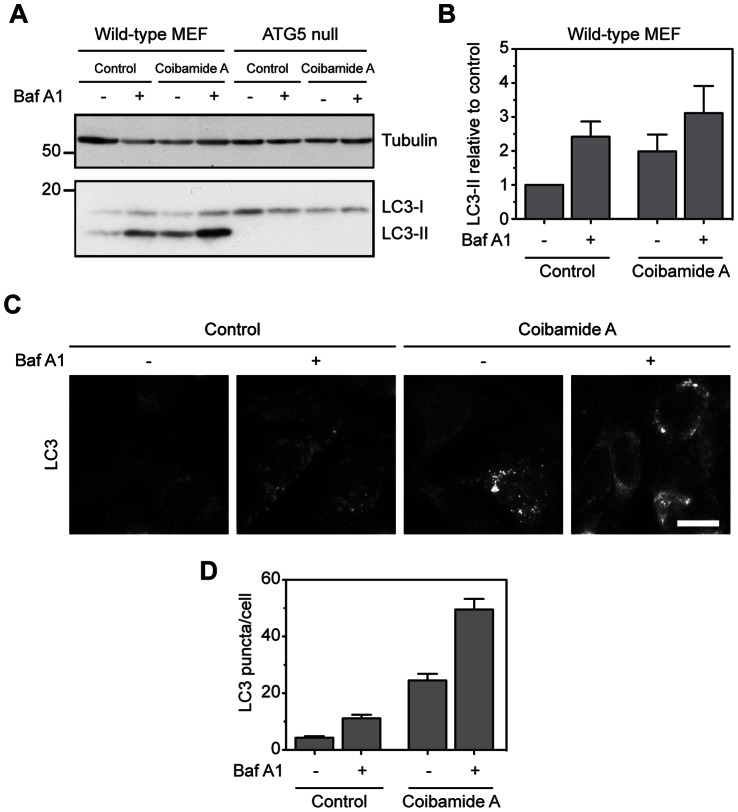
Coibamide A induces autophagy in an ATG5-dependent manner. (**A**) Immunoblot analysis of endogenous LC3 in wild-type and ATG5 null MEF cell lysates treated with vehicle (control) or coibamide A (30 nM) for 4 h, each in the presence or absence of bafilomycin (10 nM) for the final 1 h of treatment. (**B**) Quantitation of LC3-II levels in wild-type MEF cells as shown in panel A. Histogram represents mean LC3-II intensity, normalized to tubulin, from immunoblots generated in three independent experiments. (**C**) Immunocytochemistry of wild-type MEF cells treated with vehicle (control) or coibamide A (30 nM) for 4 h, each in the presence of absence of bafilomycin A1 (10 nM) for the final 1 h of treatment. Cells were stained for endogenous LC3 (red) and DAPI (blue). Scale bar, 10 µm. (**D**) Quantitation of mean LC3 puncta per cell (n = 32 cells) from three independent experiments.

The best-characterized form of macroautophagy in mammalian cells is that which is dependent on ATG 5, an essential component of the protein complex that regulates formation of the autophagosome [6]; [20]. Since an initial evaluation of coibamide A-induced responses in MEFs revealed that this cell type was also vulnerable to coibamide A-induced cell death (Fig. S1), we evaluated the potential of coibamide A to induce autophagosome accumulation in both immortalized wild-type and ATG5-null MEFs [21]. For these studies, cells were treated with or without coibamide A for 4 h in the presence or absence of bafilomycin A1 for the last hour of treatment. Under these conditions, wild-type MEFs showed a clear increase in endogenous LC3-II in response to coibamide A and this signal was enhanced further by the addition of bafilomycin A1. In contrast, endogenous LC3-II was not detected in ATG5-null MEFs in response to any of the treatment conditions (Fig. 9A and 9B). When visualized using fluorescence microscopy, control wild-type MEFs showed relatively few LC3 puncta, whereas coibamide A-treated cells showed numerous LC3 puncta per cell (Fig. 9C and 9D). Furthermore, the number of LC3 puncta per cell was enhanced when wild-type MEFs were exposed to coibamide A in the presence of bafilomycin A1 (Fig. 9C and 9D). These data indicate that coibamide A induces autophagosome formation in an ATG5-dependent manner consistent with the findings in U87-MG cells.

### Coibamide A-induced Autophagy is mTOR-independent

**Figure 10:**
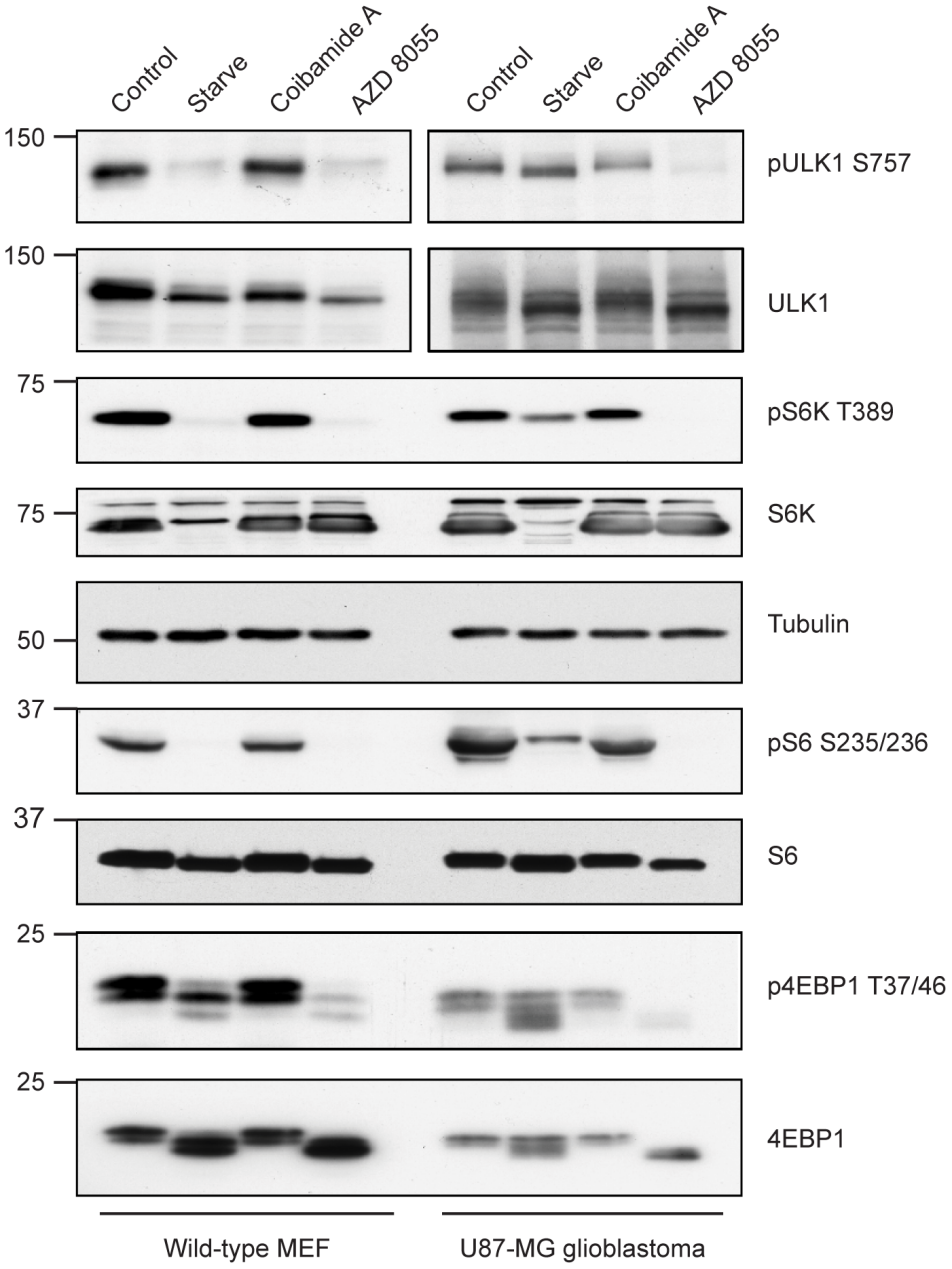
Coibamide A induces mTOR-independent autophagy. Coibamide A-induced signaling is mTOR-independent and can be distinguished from starvation- and AZD 8055-induced autophagy in MEF and U87-MG glioblastoma cells. Cells were incubated in EBSS starvation medium, or treated with or without coibamide A (30 nM) or the dual mTOR inhibitor AZD 8055 (300 nM) in standard nutrient-rich medium for 4 h. Following treatment cells were lysed and subjected to immunoblot analysis. The phosphorylation status of residues considered to be major targets of the mTOR pathway was assessed: phospho-ULK1 (Thr-757), phospho-p70 S6 Kinase (Thr-389), phospho-S6 ribosomal protein (Ser-235/236), and phospho-4E-BP1 (Thr-37/46) are shown relative to total ULK1, S6K1, S6 ribosomal protein and 4-E binding protein, respectively. Tubulin served as a loading control. Results are representative of at least three independent experiments.

To explore the molecular mechanisms underlying coibamide A-induced signaling, we compared coibamide A responses to both starvation- and pharmacologically-induced autophagy in MEF and U87-MG glioblastoma cells. For these studies we incubated cells in EBSS starvation medium for 4 h, or treated cells with either coibamide A (30 nM) or the dual mammalian Target Of Rapamycin (mTOR) complex inhibitor AZD 8055, [22],[23], (300 nM) in standard nutrient-rich medium for 4 h. Cell lysates were then analysed to determine the impact of coibamide A on the mTORC1 protein kinase complex. Firstly we looked at ULK1, the autophagy-initiating kinase that can be directly inhibited by mTOR phosphorylation. We evaluated the phosphorylation state of the major mTORC1 target site on ULK1, Ser-757, and found no decrease in phosphorylation of this residue in coibamide A-treated cells (Fig. 10). In contrast, starved and AZD 8055-treated control MEFs and U87-MG glioma cell lysates showed dephosphorylation of Ser-757 or a corresponding shift in the mobility of total ULK1, consistent with an mTOR-mediated response (Fig. 10). We also examined the downstream effectors of mTORC1 in MEFs and U87-MG cell lysates and found no decrease in the phosphorylation state of p70 S6K1 (Thr-389), S6 ribosomal protein (Ser-235/236) and 4EBP-1 (Thr-37/46) in these cells in response to coibamide A treatment (Fig. 10). Additional analyses of coibamide A-induced autophagy, relative to starvation-induced autophagy, in human SF-295 (Fig. S2)) and U251 (Fig. S3) glioblastoma cells also revealed increases in endogenous LC3-II expression with no change in the phosphorylation state of p70 S6K1 (Thr-389) or the mobility of 4EBP-1. These results indicate that coibamide A is not an inhibitor of the mTORC1 protein complex and likely regulates autophagy via an mTOR-independent signaling pathway.

### Coibamide A Induces Cell Death in the Absence of ATG5 and Intrinsic Apoptotic Activating Factor-1 (Apaf-1)

**Figure 11:**
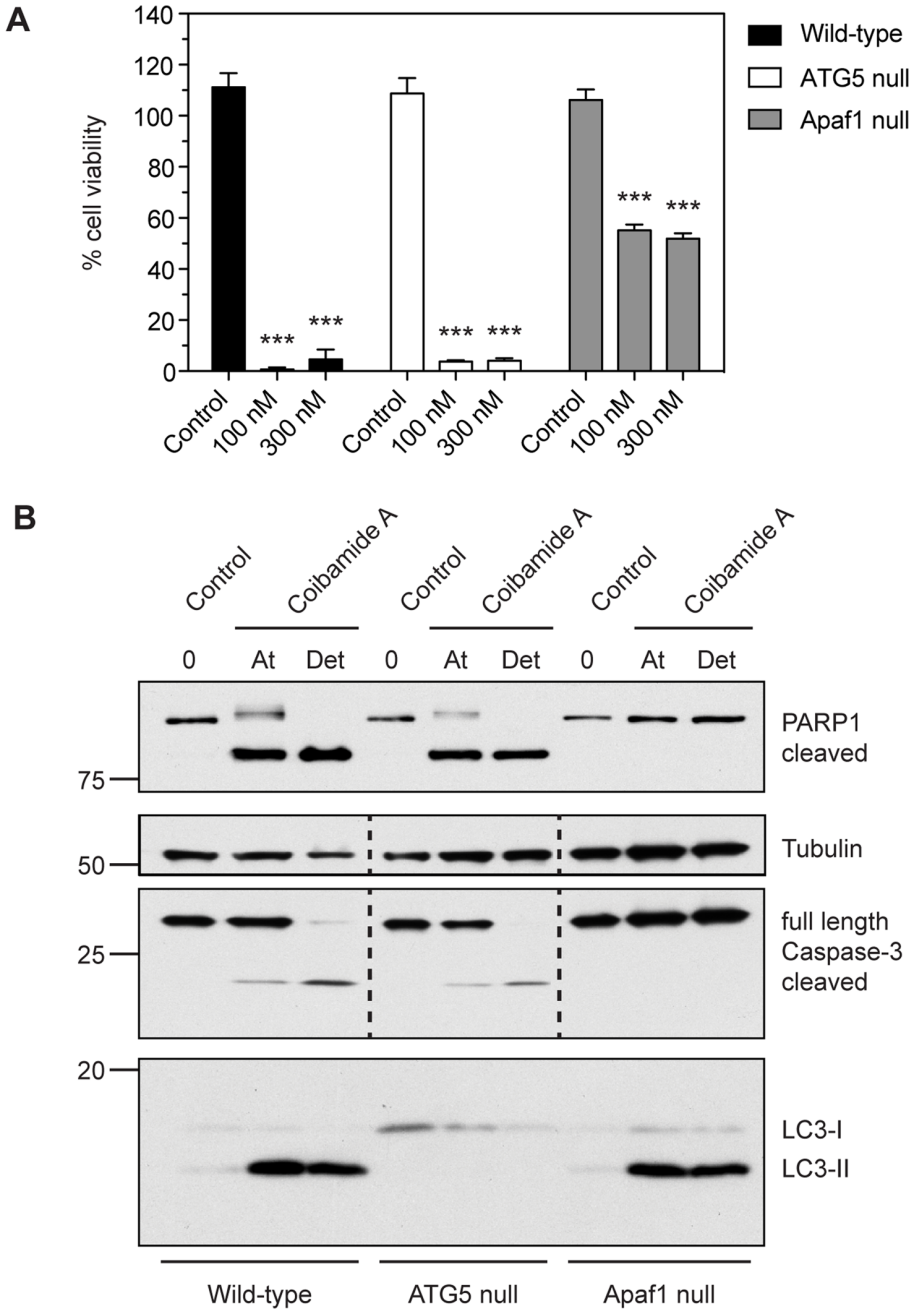
Coibamide A induces cell death in the absence of ATG5 and intrinsic Apoptotic activating factor 1 (Apaf-1). (**A**) Comparison of coibamide A-induced cytotoxicity in wild-type, ATG5 null and Apaf-1 null MEFs. MEFs cells were treated with increasing concentrations of coibamide A (0.1 to 300 nM) for 48 h. Cytotoxicity was determined by MTT assay with the viability of control cells defined as 100%. Dose-response data represent mean viability ± SE (n = 3 wells per treatment) from a comparison that was repeated in at least three independent experiments. (B) Immunoblot analysis of autophaphagy (LC3) and apoptotic (PARP1 and caspase-3) markers following treatment of wild-type, ATG5-null and Apaf-1-null MEFs with coibamide A (30 nM). Adherent and detached (Det) cells were harvested after 24 h and examined for expression of: the large 89 kDa fragment of PARP1, full length and cleaved caspase-3, LC3 isoforms I and II plus tubulin as a loading control. Immunoblot is representative of an experiment repeated at least three times with similar results.

To investigate the relationship between autophagy and coibamide A-induced cell death we compared coibamide A-induced cytotoxicity in wild-type and ATG5-null MEFs, to coibamide A-induced responses in MEFs lacking the adapter protein Apaf-1 [7]. Apaf-1-null MEFs have previously been shown to be resistant to a variety of apoptotic stimuli, as Apaf-1 is a critical component of the apoptosome complex driving the intrinsic apoptotic pathway [24]. Consistent with our findings in CNS cancer cell types and wild-type MEFs, we found coibamide A to induce time- and concentration-dependent cytotoxicity in both knockout MEF lines. Coibamide A was equally efficacious in its ability to induce cell death in wild-type and ATG5-null MEFs, with essentially no viable cells remaining after a 48 h exposure to coibamide A concentrations of 100 nM or greater (Fig. 11A). Under the same conditions, coibamide A had more limited efficacy against Apaf-1-null MEFs yet still induced significant cell death; approximately 50% of Apaf-1-null MEFs remained viable by 48 h in the presence of coibamide A (100 nM or greater) (Fig. 11A). When the attached and detached coibamide A-treated MEF populations were collected and subjected to immunoblot analysis we observed distinct patterns of coibamide A-induced cell death. An 89 kDa fragment of PARP1, corresponding to the caspase-3-cleaved form of this protein, was expressed in both wild-type and ATG5-null MEFs, with concomitant detection of cleaved caspase-3 (Fig. 11B). In addition, coibamide A-treated wild-type MEFs showed enhanced levels of endogenous LC3-II in both attached and detached cells, whereas LC3-II immunoreactivity was absent in the equivalent populations of treated ATG5-null MEFs (Fig. 11B). Coibamide A-treated Apaf-1-null MEFs, however, did show an autophagy response with enhanced levels of endogenous LC3-II in both attached and detached cell populations (Fig. 11B). We found no evidence of caspase-3 activation in these cells in response to coibamide A treatment (Fig. 11B), consistent with the pattern of caspase-independent cell death observed in U87-MG glioma cells (Fig. 4B and Fig. S4). Together these results indicate that coibamide A can induce caspase-mediated apoptotic cell death in the presence or absense of an autophagy response, and can also trigger cell death in the absence of Apaf-1.

## Discussion

Coibamide A is a marine natural product that represents a lead structure in anti-cancer drug discovery [5]. In the present study we investigated the cytotoxic potential of coibamide A against human glioblastoma cells and immortalized MEFs from genetically manipulated animals. Using several independent measures of general cytotoxicity we found that coibamide A dramatically reduced the viability of U87-MG and SF-295 glioma cells in both a concentration- and time-dependent manner. Exposure to coibamide A (100 nM or less) resulted in: a progressive decrease in the ability of cells to reduce MTT, a loss of plasma membrane integrity and detachment of cells from culture dishes. This potent nanomolar activity was lost upon linearization of the molecule, indicating the importance of the intact lariat macrocycle for inducing growth inhibitory and cytotoxic responses. Our more detailed analysis of the mechanisms by which coibamide A induces cell death showed that distinct signaling pathways can be initiated according to cell type. SF-295 glioblastoma cells underwent caspase-3 activation and apoptosis in response to coibamide A, in a pattern that was also seen in wild-type and autophagy-deficient MEFs. In contrast, U87-MG glioblastoma cells, which were approximately three-fold more sensitive to coibamide A, underwent an alternate form of cell death that was exacerbated by the presence of the caspase inhibitor Z-VAD-FMK and characterized by extensive cytoplasmic vacuolization and a lack of apoptotic features. Although it is not yet possible from this study to rule out a potentiating effect of Z-VAD-FMK at low coibamide A concentrations, further analysis of Apaf-1-null MEFs revealed that coibamide A can indeed trigger significant cell death even in the absence of the cytochrome *c*-mediated apoptotic pathway. Taken together, our studies provide the first insight into the potential of coibamide A to induce two morphologically distinct forms of cell death in a cancer cell type that is typically resistant to treatment.

A notable feature of the cellular response to coibamide A was the finding that coibamide A induces early autophagosome accumulation without inhibition of the mTOR pathway. We used a variety of approaches including analysis of the phosphorylation state of mTOR target proteins, analysis of LC3 flux in U87-MG and wild-type and mutant MEFs, plus analysis of endocytosis-mediated EGFR degradation, to conclude that this is likely a specific mTOR-independent cellular response. Autophagy is a highly regulated process that is tightly linked to the nutrient-sensing pathway of the cell [25]. During cellular stress or starvation, cells undergo a form of self-eating whereby cytoplasmic components are engulfed by double membrane-bound vesicles known as autophagosomes. Autophagosomes subsequently fuse with lysosomes, forming the autolysosome and thereby promoting the degradation of its contents by lysosomal hydrolases. This process provides both a source of energy for the cell and an alternative to the ubiquitin-proteasome system that is intended for removal of organelles and aggregated proteins [25]. Multiple signal transduction mechanisms are known to regulate autophagy and these can be broadly classified as those that signal via the mTOR pathway (mTOR-dependent), and those that do not (mTOR-independent). mTOR is a serine/threonine kinase that functions in the context of two distinct protein complexes: mTOR Complex 1 (mTORC1) and mTOR Complex 2 (mTORC2). We compared the action of coibamide A to that of two pharmacologic inducers of autophagy: rapamycin which is an effective, but incomplete, inhibitor of mTORC1 [26],[27], and AZD 8055 which inhibits both mTOR complexes [22],[23]. Although initial comparisons of rapamycin- and coibamide A-induced effects in U87-MG cells revealed increases in LC3-II and a pattern of MDC staining that were qualitatively similar, we found no evidence for inhibition of the mTOR pathway after analysis of the phosphorylation state of key residues on ULK1, p70 S6K1, S6 ribosomal protein and 4EBP-1, relative to starvation- and AZD8055-induced autophagy in human glioblastoma cells and MEFs. This lack of mTOR inhibition, together with the observation that the overall rate of autophagic flux was not changed dramatically by coibamide A, supports our observation that cytoxtoxicity was somewhat delayed in response to coibamide A. Had the energy status of the cell been compromised within hours of coibamide A exposure, indirect inhibition of the mTOR pathway could have been anticipated through activation of AMP-activated proten kinase (AMPK), the cellular sensor of energy stress [28]. Taken together, our results place coibamide A with a growing list of autophagy modulators that signal independently of mTOR. Further work will be required to determine if coibamide A acts via a known mTOR-independent pathway or via a new target.

Based on our study of ATG5 null MEFs, our results indicate that autophagy is not required for coibamide A-induced cell death even though autophagy persisted in cells dying via apoptotic or non-apoptotic mechanisms in response to treatment. This finding is consistent with the current consensus that autophagy is usually a survival response by the cell that may change the rate of cell death but rarely initiates cell death [29]. Defective autophagy has now been implicated in many chronic human diseases including cancer and neurodegenerative conditions [30],[31]. Furthermore, different cancers have varying susceptibility to the signaling pathways that are currently known to regulate mammalian autophagy. This raises the issue of whether autophagy is a positive or negative regulator of cancer cell survival [32],[33]. Since the molecular target of coibamide A is not yet known, the potential separation of autophagic and cytotoxic responses following coibamide A exposure represents a significant step, as many small molecules induce autophagy in normal and cancer cell types, but do not necessarily cause cell death. For example, rapamycin has long been considered to be a potent cytostatic natural product, rather than a cytotoxic agent [34]. Similarly, a recent comparison of rapamycin, rottlerin and three drugs approved for human use (amiodarone, niclosamide and perhexiline), highlights the fact that transient induction of autophagy under conditions of nutrient sufficiency is not harmful to the cell [35]. All five compounds were capable of stimulating mTOR-dependent autophagy but only amiodarone induced cell death, leading the authors to conclude that, in the case of amiodarone, cytotoxicity is likely a consequence of activity at cellular targets other than mTORC1 [35].

Our results demonstrate that coibamide A is a more potent cytotoxin against human glioblastoma cells than was previously appreciated [5]. As coibamide A was found to be cell cycle active [5], we exposed cells beyond the 48 h that is routinley used in the NCI in vitro therapeutic development screen to reveal a clear concentration-dependent cytotoxic response. Cell death in response to coibamide A was apoptotic in SF-295 glioma cells, yet proceeded via a non-apoptotic pathway in U87-MG glioma cells. The genetic background of U87-MG cells is classified as PTEN-deficient with normal p53 function, whereas SF-295 cells are deficient in both p53 and PTEN signaling pathways. Mutations in these two genes occur very frequently in GBM; analysis of 601 genes from 91 GBM patient samples, recently completed as part of The Cancer Genome Atlas project, showed the incidence of *TP53* and *PTEN* mutations to be 42% and 33%, respectively [4]. Of the six glioma cell lines represented in the NCI60 panel against which coibamide A was tested, four (SF-295, U251, SF-539, SNB-19) are characterized as being variant in both PTEN and p53 pathways, whereas two (SNB-75, SF-268) have variations in the p53 pathway, but normally functioning PTEN. Taken together these data suggest that coibamide A may be an effective cancer cell toxin regardless of p53 and PTEN status.

With increasing recognition of alternate cell death pathways, the inherent ability of a small molecule to induce more than one mode of cell death may be a particularly useful pharmacological property in cancer therapeutics [36]. The histone deacetylase inhibitor, suberoylanilide hydroxamic acid (SAHA), for instance, is a strong inducer of autophagy and caspase-dependent apoptotic cell death in several cell types [37],[38]. However, non-apoptotic cell death still occurs when caspase activation is blocked via pharmacologic or genetic manipulation of these cells [37],[38]. Furthermore, inhibition of autophagy can enhance both apoptotic and non-apoptotic forms of SAHA-induced cell death, defining a general protective role of autophagy in response to SAHA treatment [38]. Sanguilutine is another small molecule that has been reported to induce autophagy in human A-375 melanoma cells, with cell death mediated by a non-apoptotic caspase-independent pathway [39]. In addition, the marine algal toxin, yessotoxin, has been shown to induce autophagy and cell death through both apoptotic and non-apoptotic pathways in numerous model cell systems [40]. Together with coibamide A, these small molecule cytotoxins may be useful pharmacological tools for studying non-apoptotic cell death particularly in cancer cells.

Natural products from terrestrial and marine organisms have a track record of potent cancer cell toxicity that has led to the development of several as clinical candidates [41]. Notable historical examples from marine cyanobacteria are anti-tubulin agents, the cryptophycins, dolastatins 10 and 15 and curacin A [41],[42]. Marine cyanobacteria have continued to be a prolific source of cytotoxic depsipeptides applicable to cancer research and pharmaceutical development [43],[44]. The peptidic molecular structure of coibamide A is stabilized by a high degree of methylation that favors cell membrane permeability and proteolytic stability. Our data also indicate that cyclization of the peptide is a critical determinant of the cellular response to coibamide A, with two linear forms showing no cytotoxicity to glio- and neuroblastoma cells. This phenomenon has guided ongoing efforts to chemically synthesize biologically active analogs of coibamide A. Our findings are also in contrast to the example of desmethoxy-majusculamide C (DMMC), a cyclic depsipeptide isolated from a Fijian marine cyanobacterium [45]. DMMC is a potent disrupter of the actin cytoskeleton that exhibits nanomolar cytotoxicity in linear form that is equivalent to the activity of the intact cyclic natural product.

In summary, coibamide A is an unusual cyclopeptide that potently inhibits cell growth and induces cell death in human glioblastoma cells. Although the molecular target of coibamide A has still to be established, our findings provide insight into a new marine natural product structure that may be an invaluable tool for the study of both mTOR-independent autophagy and cell death signaling in apoptosis-resistant cancer cells. Our clarification of the cytotoxic potential of coibamide A, together with the identification of MEFs as a sensitive cell type, will facilitate further evaluation of coibamide A as a potential lead compound.

## Supporting Information

Figure S1 **Coibamide A is cytotoxic to wild-type MEFs.** Concentration-response profile for coibamide A-induced cytotoxicity in wild-type MEFs. Cells were treated with increasing concentrations of coibamide A (0.1 to 300 nM) for 48 h. Cytotoxicity was determined by MTT assay with the viability of control cells defined as 100%. Dose-response data represent mean viability ± SE (n = 3 wells per treatment) from a comparison that was repeated in at least four independent experiments.
(TIF)

Figure S2 **Coibamide A induces mTOR-independent autophagy in human SF-295 glioblastoma cells.** Human SF-295 glioblastoma cells were incubated in EBSS starvation medium, or treated with or without coibamide A (30 nM) in standard nutrient-rich medium for 4 h. Following treatment cells were lysed and subjected to immunoblot analysis. (A) Immunoblot analysis of LC3 expression, phospho-p70 S6 Kinase (Thr-389) relative to total S6K1, and 4-E binding protein. (B) LC3 expression is diminished in SF-295 cells after 4 h incubation in starvation medium, relative to LC3 expression in vehicle (control) or coibamide A (30 nM)-treated cells in standard nutrient-rich medium. GAPDH served as a loading control. Results are representative of at least four independent experiments.
(TIF)

Figure S3 **Coibamide A induces mTOR-independent autophagy in human U251 glioblastoma cells.** Human U251 glioblastoma cells were incubated in EBSS starvation medium, or treated with or without coibamide A (30 nM) in standard nutrient-rich medium for 4 h. Following treatment cells were lysed and subjected to immunoblot analysis. (A) Immunoblot analysis of LC3 expression, phospho-p70 S6 Kinase (Thr-389) relative to total S6K1, and 4-E binding protein. GAPDH served as a loading control. (B) Immunoblot analysis of endogenous LC3 in U251 cells treated with vehicle (control), coibamide A (30 nM), or EBSS starvation medium for 4 h, each in the presence or absence of bafilomycin (10 nM) for the final 1 h of treatment. GAPDH served as a loading control. Results are representative of three independent experiments.
(TIF)

Figure S4 **Expression of apoptotic markers in wild-type MEFs and human U87-MG glioblastoma cells in response to coibamide A treatment.** Immunoblot analysis of PARP1 and caspase-3 in wild-type MEFs and U87-MG cells after treatment with coibamide A (30 nM). Adherent and detached (Det) cells were harvested 24 h (MEFs) and 72 h (U87-MG) after treatment and examined for expression of the large 89 kDa fragment of PARP1, full length and cleaved caspase-3, and GAPDH as a loading control. Immunoblot is representative of an experiment repeated at least three times with similar results.
(TIF)
