# Supplementary figures and images for: Coibamide A Induces mTOR-Independent Autophagy and Cell Death in Human Glioblastoma Cells
Source: PLoS One. 2013 Jun 6;8(6):e65250. doi: 10.1371/journal.pone.0065250 (PMC3675158; doi:10.1371/journal.pone.0065250)

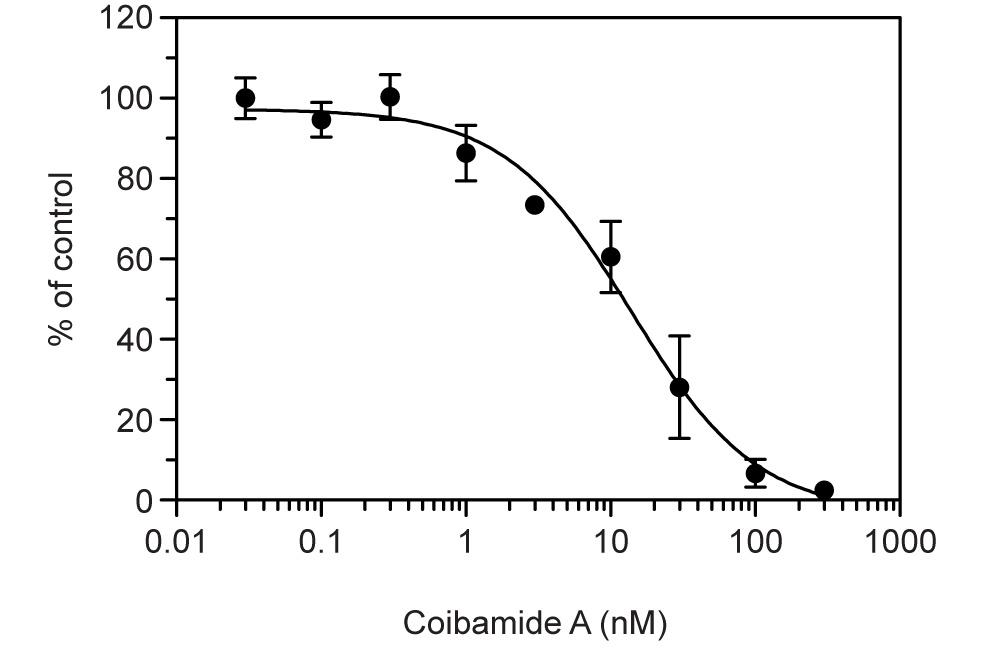

Supplement: Figure S1 — Coibamide A is cytotoxic to wild-type MEFs. Concentration-response profile for coibamide A-induced cytotoxicity in wild-type MEFs. Cells were treated with increasing concentrations of coibamide A (0.1 to 300 nM) for 48 h. Cytotoxicity was determined by MTT assay with the viability of control cells defined as 100%. Dose-response data represent mean viability ± SE (n = 3 wells per treatment) from a comparison that was repeated in at least four independent experiments. (TIF) [file pone.0065250.s001.tif]

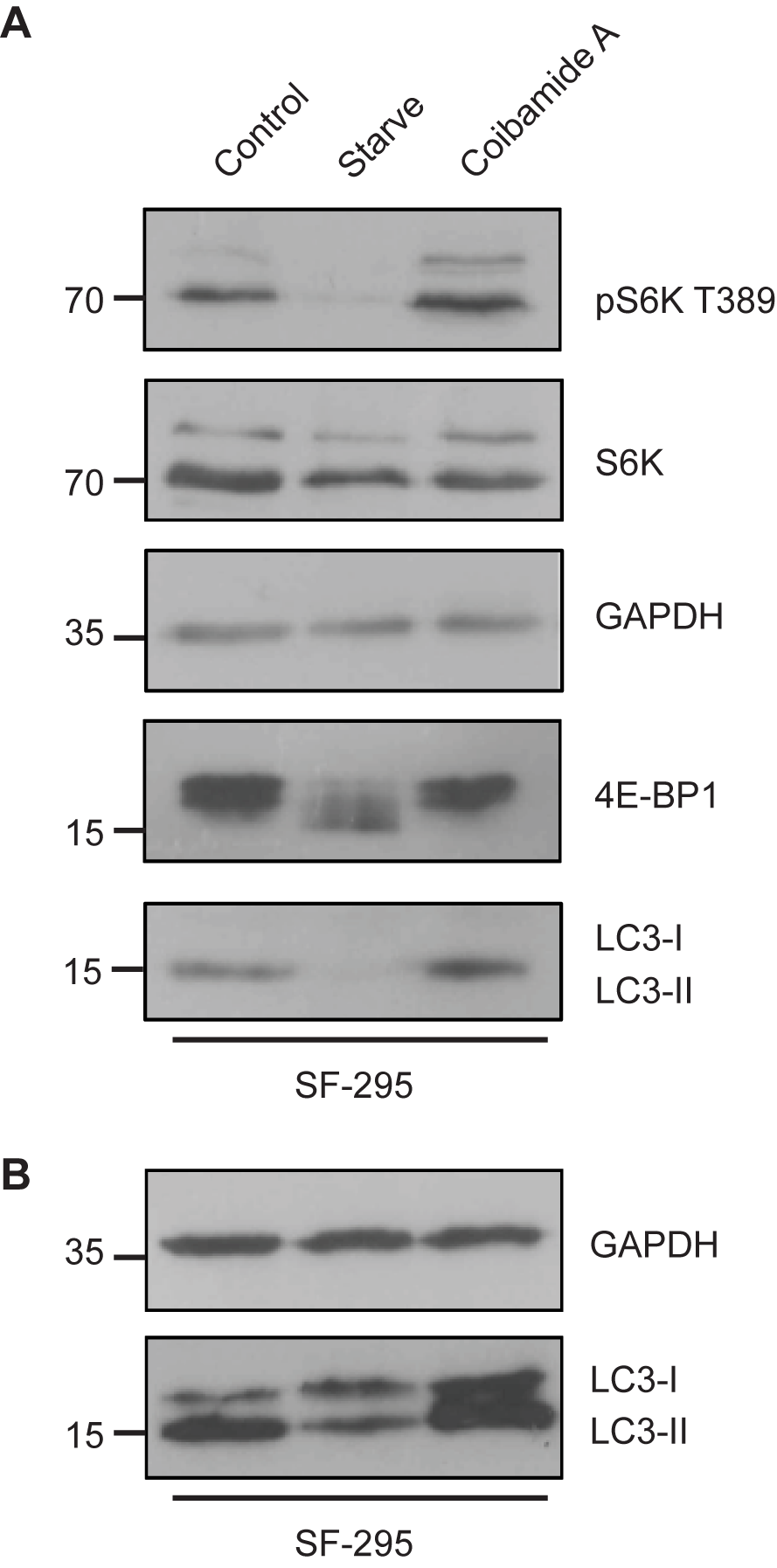

Supplement: Figure S2 — Coibamide A induces mTOR-independent autophagy in human SF-295 glioblastoma cells. Human SF-295 glioblastoma cells were incubated in EBSS starvation medium, or treated with or without coibamide A (30 nM) in standard nutrient-rich medium for 4 h. Following treatment cells were lysed and subjected to immunoblot analysis. (A) Immunoblot analysis of LC3 expression, phospho-p70 S6 Kinase (Thr-389) relative to total S6K1, and 4-E binding protein. (B) LC3 expression is diminished in SF-295 cells after 4 h incubation in starvation medium, relative to LC3 expression in vehicle (control) or coibamide A (30 nM)-treated cells in standard nutrient-rich medium. GAPDH served as a loading control. Results are representative of at least four independent experiments. (TIF) [file pone.0065250.s002.tif]

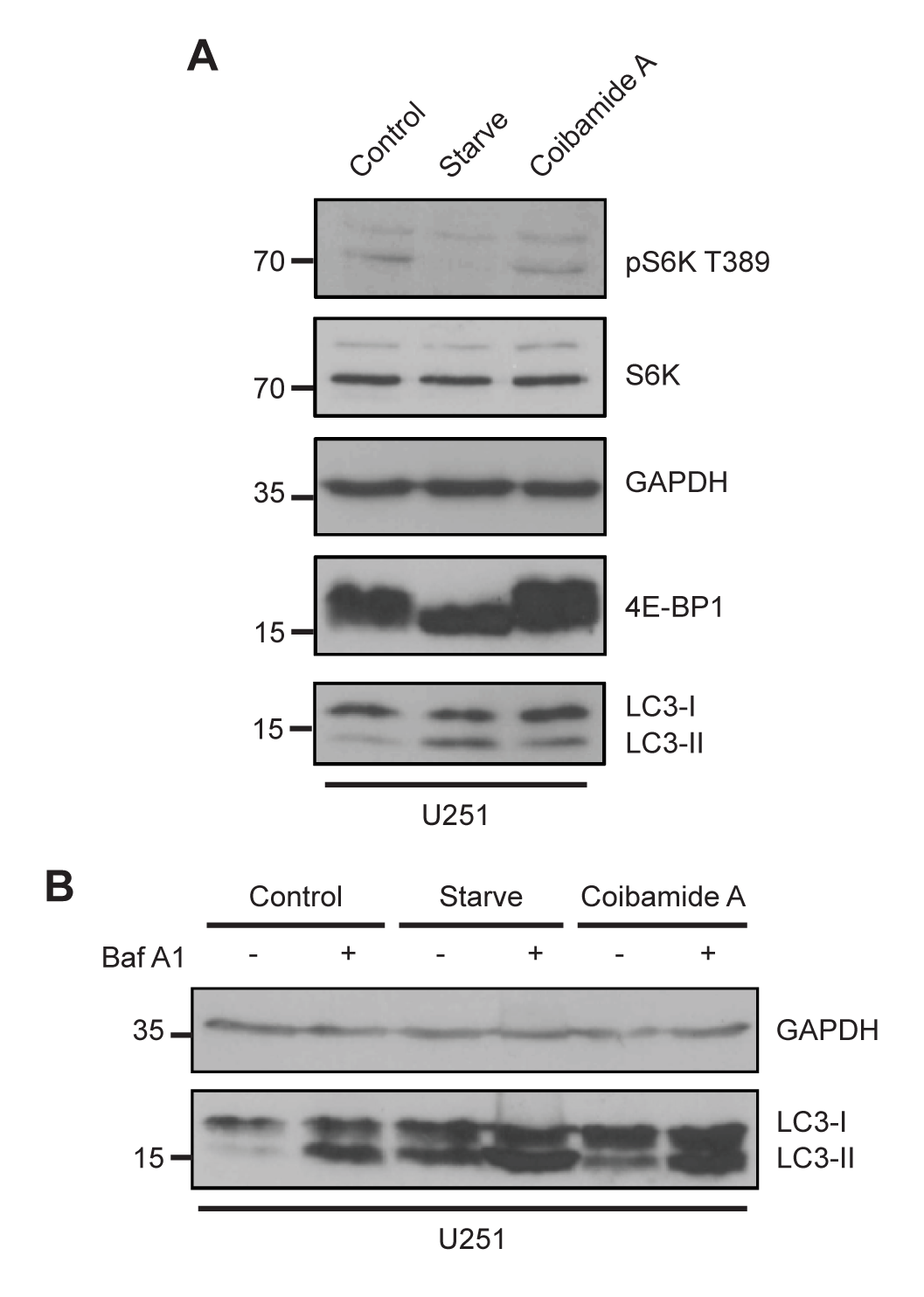

Supplement: Figure S3 — Coibamide A induces mTOR-independent autophagy in human U251 glioblastoma cells. Human U251 glioblastoma cells were incubated in EBSS starvation medium, or treated with or without coibamide A (30 nM) in standard nutrient-rich medium for 4 h. Following treatment cells were lysed and subjected to immunoblot analysis. (A) Immunoblot analysis of LC3 expression, phospho-p70 S6 Kinase (Thr-389) relative to total S6K1, and 4-E binding protein. GAPDH served as a loading control. (B) Immunoblot analysis of endogenous LC3 in U251 cells treated with vehicle (control), coibamide A (30 nM), or EBSS starvation medium for 4 h, each in the presence or absence of bafilomycin (10 nM) for the final 1 h of treatment. GAPDH served as a loading control. Results are representative of three independent experiments. (TIF) [file pone.0065250.s003.tif]

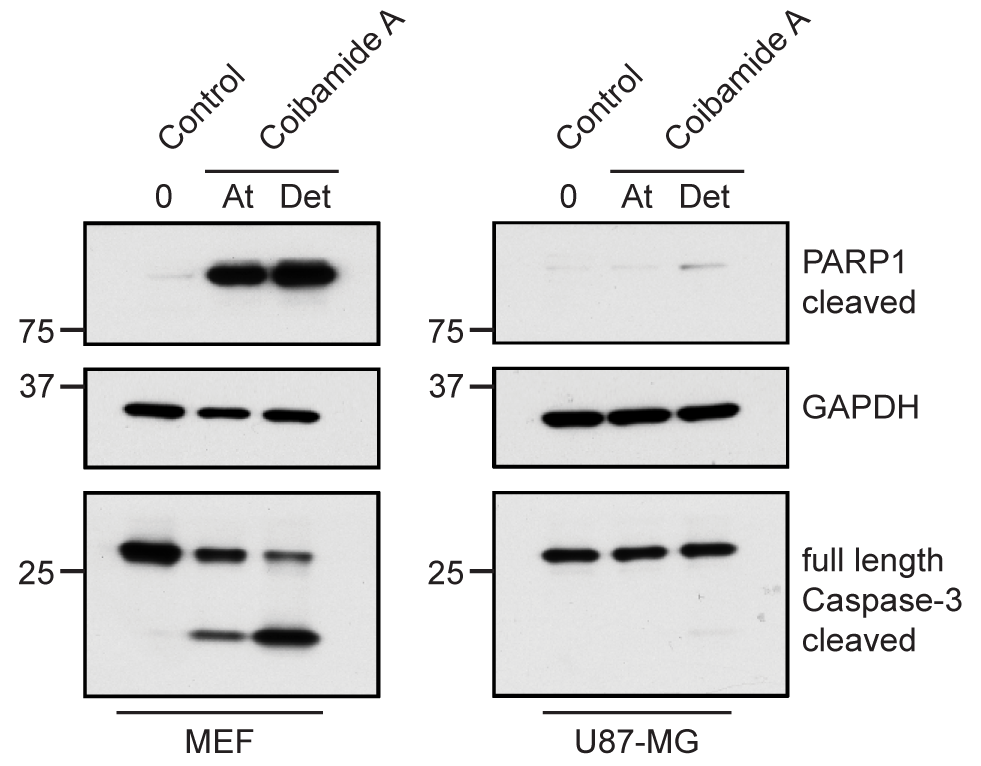

Supplement: Figure S4 — Expression of apoptotic markers in wild-type MEFs and human U87-MG glioblastoma cells in response to coibamide A treatment. Immunoblot analysis of PARP1 and caspase-3 in wild-type MEFs and U87-MG cells after treatment with coibamide A (30 nM). Adherent and detached (Det) cells were harvested 24 h (MEFs) and 72 h (U87-MG) after treatment and examined for expression of the large 89 kDa fragment of PARP1, full length and cleaved caspase-3, and GAPDH as a loading control. Immunoblot is representative of an experiment repeated at least three times with similar results. (TIF) [file pone.0065250.s004.tif]
